# Supplementary material for: Overexpression of p-4EBP1 associates with p-eIF4E and predicts poor prognosis for non-small cell lung cancer patients with resection
Source: PLoS One. 2022 Jun 23;17(6):e0265465. doi: 10.1371/journal.pone.0265465 (PMC9223369; doi:10.1371/journal.pone.0265465)
Supplement: Attachment — Submitted filename: Response to Reviewers.docx [file pone.0265465.s001.docx]

**Rebuttal letter**

Dear Editors and reviewer,

Thank you very much for the timely review on our manuscript entitled “Overexpression of p-4EBP1 associates with p-eIF4E and predicts poor prognosis for non-small cell lung cancer patients with resection” [PONE-D-21-09571], we believe these comments would be of great help to improve the manuscript. We have carefully revised the manuscript according to these comments, our point-by-point response to these suggestions are as following:

**Journal Requirements:**

1. Please ensure that your manuscript meets PLOS ONE's style requirements, including those for file naming. The PLOS ONE style templates can be found at

https://journals.plos.org/plosone/s/file?id=wjVg/PLOSOne_formatting_sample_main_body.pdf and

https://journals.plos.org/plosone/s/file?id=ba62/PLOSOne_formatting_sample_title_authors_affiliations.pdf

**Response:** We have ensured that our manuscript meets PLOS ONE's style requirements, including those for file naming.

2. Please provide additional details regarding participant consent. In the ethics statement in the Methods and online submission information, please ensure that you have specified what type you obtained (for instance, written or verbal, and if verbal, how it was documented and witnessed). If your study included minors, state whether you obtained consent from parents or guardians. If the need for consent was waived by the ethics committee, please include this information.

**Response:** We have provided additional details regarding participant consent both in Chinese and English versions.

3. We note that the grant information you provided in the ‘Funding Information’ and ‘Financial Disclosure’ sections do not match. When you resubmit, please ensure that you provide the correct grant numbers for the awards you received for your study in the ‘Funding Information’ section.

**Response:** We have completed the modification in the "Funding Information" section and have confirmed that the grant numbers are correct. The "Funding Information" section is described below:

The work was supported by National Natural Science Foundation of China (grant No. 81703009, 81773218, and 81972838), the Natural Science Foundation of Hunan Province (grant No. 2017JJ3457), Hunan Provincial Innovation Foundation for Postgraduate (grant No. CX20210373), and the Fundamental Research Funds for the Central Universities of Central South University (grant No. 2021zzts1045).

4. Thank you for stating the following in the Acknowledgments Section of your manuscript:

The work was supported by grants of National Natural Science Foundation of China (grant No. 81703009, 81773218, and 81972838) and The Natural Science Foundation of Hunan Province (grant No. 2017JJ3457).

We note that you have provided additional information within the Acknowledgements Section that is not currently declared in your Funding Statement. Please note that funding information should not appear in the Acknowledgments section or other areas of your manuscript. We will only publish funding information present in the Funding Statement section of the online submission form.

Please remove any funding-related text from the manuscript and let us know how you would like to update your Funding Statement. Currently, your Funding Statement reads as follows:

The author(s) received no specific funding for this work.

Please include your amended statements within your cover letter; we will change the online submission form on your behalf.

**Response:** Thank you very much for your suggestion. We were so sorry to confuse the editor to make Funding Statement “The author(s) received no specific funding for this work” by mistake in the first submission. We have modified the "Funding Information" in the revised manuscript and made a detailed and correct description in Funding Statement in our cover letter. The Funding Statement is described below:

The work was supported by National Natural Science Foundation of China (grant No. 81703009, 81773218, and 81972838), the Natural Science Foundation of Hunan Province (grant No. 2017JJ3457), Hunan Provincial Innovation Foundation for Postgraduate (grant No. CX20210373), and the Fundamental Research Funds for the Central Universities of Central South University (grant No. 2021zzts1045).

5. We note that you have stated that you will provide repository information for your data at acceptance. Should your manuscript be accepted for publication, we will hold it until you provide the relevant accession numbers or DOIs necessary to access your data. If you wish to make changes to your Data Availability statement, please describe these changes in your cover letter and we will update your Data Availability statement to reflect the information you provide.

**Response:** Thanks for your suggestion. We have described our changes to our Data Availability statement in our cover letter. The Data Availability statement is described below: All data relevant to the study are included in the article.

We were so sorry to confuse the editor to make Data Availability Statement “All relevant data are within the manuscript and its Supporting Information files.” by mistake in the additional information part of the submission system. We have modified it to “All relevant data are within the manuscript”.

6. PLOS requires an ORCID iD for the corresponding author in Editorial Manager on papers submitted after December 6th, 2016. Please ensure that you have an ORCID iD and that it is validated in Editorial Manager. To do this, go to ‘Update my Information’ (in the upper left-hand corner of the main menu), and click on the Fetch/Validate link next to the ORCID field. This will take you to the ORCID site and allow you to create a new iD or authenticate a pre-existing iD in Editorial Manager. Please see the following video for instructions on linking an ORCID iD to your Editorial Manager account: https://www.youtube.com/watch?v=_xcclfuvtxQ

**Response:** Thanks a lot for your help. We have updated our information and authenticated the pre-existing iD in Editorial Manager following your guides.

7. Your ethics statement should only appear in the Methods section of your manuscript. If your ethics statement is written in any section besides the Methods, please delete it from any other section.

**Response:** We have completed the modification as required in the revised manuscript.

8. We note you have included a table to which you do not refer in the text of your manuscript. Please ensure that you refer to Table 4 in your text; if accepted, production will need this reference to link the reader to the Table.

**Response:** We have completed the modification as required in the revised manuscript.

**Additional Editor Comments:**

Based on the reviewer´s comments and based on the editor´s suggestions a major revision is required. In addition to the comments from referee one also the work by Gantenbein Nadine et al on eIF6 in lung cancer should be discussed.

**Response:** Thank you for your advice. We have revised the manuscript according to the editor´s suggestions and discussed the work by Gantenbein Nadine et al on eIF6 in lung cancer in detail in the revised manuscript.

**Reviewer :**

1. Smoking status, PD-L1 expression level, and the information of oncogenic driver alterations, such as EGFR mutation and ALK fusion, would be useful to show the importance of p-4EBP1 and phospho-eIF4E expression levels in clinical field.

**Response:** We thank the reviewer for the comments. The reviewer mentioned three modules here: smoking status, PD-L1 expression level and the information of oncogenic driver alterations, such as EGFR mutation and ALK fusion, which are useful to emphasis the importance of p-4EBP1 and phospho-eIF4E expression levels in clinical field.

**Smoking status** Just as the reviewer highlighted the importance of relationship between smoking and the expression of p-4EBP1 and p-eIF4E, accumulating evidence has indicated nicotine is associated with mTOR pathway activation. For example, nicotine can promote the proliferation of human papillomavirus (HPV)-immortalized cervical epithelial cells H8 cells by activating Akt/mTOR pathway and inducing 4EBP1 phosphorylation [1]. Furthermore, nicotine and tobacco carcinogens can quickly activate Akt and mediate carcinogenesis [2]. In the present study, we would have analyzed the relationship between smoking status and the expression of p-4EBP1 and p-eIF4E, unfortunately, the patient's smoking status was not included in the original follow-up data. We have been aware of this problem and following up one by one to supplement the relevant information, but it is inevitable deficient and has not been finished yet since our samples were collected pretty much long time ago. We’ll keep on going to complete the data and take smoking status into account when we establish new clinicopathological data in the future.

**PD-L1 expression level** Our previous study has detected the expression level of PD-L1 in non-small cell lung cancer (NSCLC) and found that PD-L1 was highly expressed in NSCLC tissues, which is related to tumor lymph node metastasis and poor prognosis of patients [3]. At present, there is no research report on the association between the expression of PD-L1, p-4EBP1 and p-eIF4E. Thanks for the suggestion of the reviewer, we will focus on this new field and further explore the internal relationship between PD-L1, p-4EBP1 and p-eIF4E in the follow-up work.

**The information about changes in carcinogenic drivers, such as EGFR mutation and ALK fusion** In our current study, none of the patients had EGFR mutation and ALK fusion detected and targeted therapy. Firstly, EGFR gene mutation and ALK fusion mutation strategy has not been received great importance and popularity many years ago. Secondly, the majority patients from remote mountainous areas could not afford the expensive costs of inspection and targeted treatment. Nowadays, not only EGFR mutation and ALK fusion detection has become a part of routine testing, but also other relevant carcinogenic drivers such as ROS1, MET, BRAF, TP53 and so on have been suggested to be detected in our department. Above all, thanks a lot for the reviewers’ suggestion, which is very important and helpful for our follow-up research.

[1] Chen L et al. eIF4E is a critical regulator of human papillomavirus (HPV)-immortalized cervical epithelial (H8) cell growth induced by nicotine. Toxicology. 2019; 419:1-10.

[2] West KA et al. Rapid Akt activation by nicotine and a tobacco carcinogen modulates the phenotype of normal human airway epithelial cells. J Clin Invest. 2003;111(1):81-90.

[3] Zheng H et al. Co-expression of PD-L1 and HIF-1α predicts poor prognosis in Patients with Non-small Cell Lung Cancer after surgery. J Cancer. 2021;12(7):2065-2072.

2. A variety of previous studies already showed the association of several eukaryotic initiation factors (eIF) with tumor progression and chemo-resistance. Therefore, to emphasize the clinical importance of p-4EBP1 and phospho-eIF4E expression in NSCLC, analyzing the disease-free survival would be informative because most of cases could have operability.

**Response:** We thank the reviewer for the comments. Based on a meta-analysis, we know that cancer patients with lower p-4EBP1 expression had better 3-year and 5-year disease-free survival [1]. Clear cell renal cell carcinoma patients whose tumors stained positive for p-4EBP1 had a higher disease-free survival (DFS) compared to patients whose tumors were negative [2]. However, there are few reports on the relationship between p-eIF4E and disease-free survival. In our present study, considering that the endpoint of disease-free survival is difficult to record, and that patients with cancers often have complications, which will interfere with the judgment of disease-free survival rate, we did not analyze the relationship between the p-4EBP1, p-eIF4E and disease-free survival, but tended to analyze whether the two proteins related to the overall survival in NSCLC. Of course, We will further improve the follow-up data and analyze the association between the expression of these two proteins and disease-free survival rate in the future.

[1] Zhang T et al. Meta-analysis of the prognostic value of p-4EBP1 in human malignancies. Oncotarget. 2017;9(2):2761-2769.

[2] Campbell L et al. Phospho-4e-BP1 and eIF4E overexpression synergistically drives disease progression in clinically confined clear cell renal cell carcinoma. Am J Cancer Res. 2015;5(9):2838-48.

3. In table2, sample number of pathological grades ‘Well’ is too small to use Chi-square test. The reviewer recommends to analyze them by Fisher-exact test.

**Response:** We thank the reviewer for the suggestion. We have re-analyzed the relationship between p-4EBP1 and p-eIF4E protein expression and pathological grade by Fisher-exact test in the revised manuscript.

4. The abnormal activity of eIF complexes triggered by upstream signaling pathways is detected in many tumors, and eIFs can be a promising therapeutic target for various types of cancers. In NSCLC, eIF2β and eIF6 were shown as prognosis indicators and promising therapeutic targets (Cancer Sci. 2018 Jun;109(6):1843-1852. Eur J Cancer. 2018 Sep; 101:165-180.). The reviewer highly recommends to refer these papers and discuss the importance of the abnormal activity of eIF complexes in NSCLC.

**Response:** We thank the reviewer for the suggestion. We have studied those two papers and discussed the importance of the abnormal activity of eIF complexes in NSCLC in the revised manuscript.

Sincerely yours,

Qiuyuan Wen, MD/Ph. D

Department of Pathology of the Second Xiangya Hospital, Central South University

139 Ren Min Road, Changsha, Hunan 410011, China
